# The Diagnosis and Treatment of Functional Disorders of the Stomach

**Published:** 1889-12

**Authors:** Frederick P. Henry

**Affiliations:** Physician to the Philadelphia and to the Jefferson Medical College Hospitals


### THE DIAGNOSIS AND TREATMENT OF FUNCTIONAL DISORDERS OF THE STOMACH.[1]

By FREDERICK P. HENRY, M. D.,

Physician to the Philadelphia and to the Jefferson Medical College Hospitals.

THE distinction between functional and organic affections is by no means sharp. They encroach on each other in such a manner that the imaginary line which separates them is remarkable for its sinuosities, and here and there is so faint as scarcely to be traced. In both these respects it resembles the lines of demarcation, the so-called sutures, which both separate and unite certain cranial bones. These statements, while true in general, are of particular application to the diseases of the stomach, of which the disorders of function are generally, sooner or later, associated with some organic defect. Thus, disorders of secretion are frequently accompanied with some impairment of motor power, or even with some alteration in the secretory tissue.

Bearing in mind the difficulties inherent in the subject of functional disorders of the stomach, I will begin by stating that they are all included under the heads of disordered secretion, disordered motion, and impaired innervation. It is self-evident that these three classes of affections are not only closely associated, but interdependent. Like the primary three colors, they give rise, by their blending, to various affections in reality composite or secondary, but yet of primary importance.

My remarks upon these three modes of disordered function must be very brief and imperfect, for an elaborate discussion of either one of them would occupy more than the time allotted me.

To make myself thoroughly understood, I am obliged to make some preliminary remarks on the chemistry of normal digestion. It is well known that pepsin will not manifest its digestive power except in the presence of an acid, concerning the nature of which there has been great difference of opinion. It has been variously regarded by men of the highest attainments in chemistry and physiology, as phosphoric, sarco-lactic, lactic and hydrochloric; but, little by little, the first two were positively excluded from the discussion, which became limited to lactic and hydrochloric acids. For many years physiologists were at variance concerning these two acids, one set regarding lactic as undoubtedly the acid of the gastric juice, while another was equally positive that this acid could be no other than hydrochloric. For example, I myself was taught, by so great a physiologist as the late

1. Read October 9, 1889, in the Philadelphia County Medical Society.

Professor John C. Dalton, that the acid of this gastric juice was lactic. The cause of these discrepancies is now plainly manifest, for during stomachal digestion the reaction of the gastric contents is at one stage due to lactic, at another to hydrochloric acid. In fact, normal digestion, considered with reference to the presence of these acids, may be divided into three stages. During the first, which lasts about half an hour, lactic acid is present alone; later, lactic and hydrochloric acids are present simultaneously; and, finally, there comes a stage when the only acid present is hydrochloric. The last stage may begin half an hour after eating, or, at the latest, an hour thereafter—*i. e.*, in health, for in disease the duration of these three stages may be variously modified. The most important period of digestion is undoubtedly the last, for it is well established that pepsin acidified with hydrochloric acid digests more actively than when acidified with lactic. In fact, lactic acid is an adventitious product of the fermentation of carbohydrates, and is always found in the first stages of the digestion of a mixed diet—meat, bread, potatoes, etc.—but if egg-albumin alone be administered to a fasting animal, and the contents of the stomach examined shortly afterward, hydrochloric will be the only acid detected.

The examination of the contents of the fasting stomach, which would long ago have settled this important physiological question, has only recently been undertaken. In the first place, it disproved the prevalent medical belief that the fasting stomach is empty. Rosin[1] introduced a soft rubber tube into the stomachs of a number of individuals free from gastric disease, the time chosen for the experiments being the early morning hours before breakfast. In only two out of forty-four such experiments did he fail to obtain a sample of gastric juice, the amount of which in the forty-two positive cases varied between three and ten cubic centimetres. In thirty-one of these forty-two cases the fluid thus obtained contained free hydrochloric acid. The question whether the fasting stomach contains pepsin received a positive answer in all the cases, six in number, in which the secretion was submitted to the digestive test. The important question whether lactic acid is present in the juice of the fasting stomach received a negative answer. In nearly all the cases the secretion gave the well-known biuret reaction, indicating the presence of peptone; but as this reaction is also given by the saliva, as well as by the nasal and pharyngeal mucus, it is reasonable to suppose that the peptone was swallowed with these secretions.

Now let us pause and consider what light these physiological facts throw on the disorders of gastric function.

1. Dr. Heinrich Rosin: *Deutsche Med. Wochenschrift*, November 22, 1888.

In the first place, it is readily understood how the first, or lactic acid, stage of digestion may encroach upon the second and third stages of that process, but the question with which we are now concerned is whether this excessive lactic acidity gives rise to symptoms. There can, I think, be no doubt that such a condition is the chief cause of *pyrosis*. I do not go as far as Bourget,[1] who considers it the sole cause of that annoying symptom. He was led to this conclusion by finding that he could not produce pyrosis in his own person by swallowing two glasses of water containing five per cent. of HCl, but that it was experienced a few minutes after taking the same amount of water containing one per cent. of lactic acid with a trace of butyric acid. Pyrosis is, undoubtedly, a symptom of excessive lactic acid fermentation, and the earlier it appears after the ingestion of food the more certainly is it due to this cause. It is also a symptom of undue acidity of the gastric juice, or hyperacidity, as it is now called, it being always understood where this term is used that the acid in excess is HCl. The term *pyrosis hydrochlorica* has been applied to this condition. Finally, pyrosis is not necessarily due to an excess either of HCl or lactic acid, but may even make its appearance when the gastric juice is of subnormal acidity. It may be, in short, one of the symptoms of nervous dyspepsia, in which affection the gastric nerves are so hypersensitive as to suffer during a digestion which is, in other respects, normal.

By hyperacidity is meant, as the term implies, that the gastric juice, which may be of normal or increased amount, contains an excessive amount of HCl. This condition is not to be confounded with hypersecretion, which means that the gastric juice is secreted in excessive amount during the intervals of digestion. As I have stated elsewhere, the former consists in a too liberal response to a physiological summons, while the latter is gratuitous. Hyperacidity occurs most frequently in connection with gastric ulcer, and, therefore, scarcely comes within the range of our subject. Hypersecretion, on the other hand, is a mere exaggeration of the normal processes, for—as demonstrated by the researches of Rosin, to which I have already alluded—the fasting stomach contains an appreciable amount of gastric juice. The limits within which the secretion of gastric juice, by a fasting stomach, may be considered physiological, are, as yet, unknown. It is certain, however, that the functional gastric disorder known as hypersecretion constitutes a well-marked pathological condition. Its principal symptoms are increased thirst, acid eructations, nocturnal attacks of epigastric pain, and dilatation of the stomach. The diges-

1. *Revue Méd. de la Suisse Romande*, November 12, 1888.

tion of carbo-hydrates is greatly impaired, while that of the albuminous substances proceeds with abnormal vigor. Positive proof of this condition is afforded by the sound employed in the following manner: The stomach is thoroughly washed out in the evening, and the patient directed to fast until the following morning, when the sound is again introduced. If hypersecretion exists, a quantity of fluid, varying from eight ounces to a pint, may be withdrawn. This fluid is colorless or slightly greenish, of viscid consistence, strongly acid, the acid being hydrochloric, and digests albumin rapidly. In a word, it is gastric juice.

Hypersecretion is probably always caused by disordered gastric innervation. It occurs in two forms, the one continuous, the other paroxysmal. The latter often coincides with the *crises gastriques* of tubes; and in such cases the neurotic character of the affection is plainly evident.

A knowledge of the absorbent power of the stomach is of diagnostic value, and may be obtained by the following test, first employed by Penzoldt and Faber[1] in 1882. Three grains of potassium iodide are administered in a gelatine capsule, and the saliva, which has been previously shown to be free from iodine, is tested every minute for this substance. In dealing with a healthy and empty stomach, the first iodine reaction will be obtained in from six and one-half minutes to eleven minutes. If the drug be administered immediately after a meal, the iodine reaction will not appear until from twenty to thirty, and may even be delayed until forty-five, minutes have elapsed. By this means it has been shown that in various pathological conditions, such as cancer, ulcer, and chronic catarrh, absorption from the gastric mucosa is retarded. It can be readily understood that, in certain cases, a diminished absorption from this mucous membrane may be highly conservative. Litten[2] has demonstrated, by the test just described, a dimished power of absorption in cases of atrophy of the mucous membrane of the stomach. Now, in many such cases, especially in the forms associated with pyloric stenosis and consecutive dilatation, the stomach may contain in its fermenting and putrifying contents an amount of ptomaines sufficient, if absorbed, to cause death by acute poisoning.

The usual precautions are to be taken in the administration of gelatine capsules, *i. e.*, they should not be given to a patient who is taking either alcohol or tannic acid, as both these substances render the gelatine insoluble.

1. *Berlin. Klin. Wochenschrift, No.* 21, 1882.
2. *Deutsche Med. Wochenschrift,* Nov. 22, 1888.

It is often of diagnostic value to obtain some idea of the motor power of the stomach, which some authorities are inclined to regard as the most important function of that viscus. Until quite recently, the only method of acquiring such information was that introduced by Leube, and consisted in the introduction of the stomach-tube seven hours after a meal, *i. e.*, at a time when the food should have passed into the intestine. The presence of food at such a time would indicate an impairment of motor power, and the degree of the latter would be in direct ratio to the quantity of the former. This method is crude, and inapplicable to most clinical purposes.

The peculiar properties of salol led Ewald and Sievers to employ it for testing the motor function of the stomach. This drug remains insoluble and undecomposed in the stomach, but splits up into carbolic and salicylic acids when it comes in contact with the alkaline secretions of the intestine. The salicylic acid is excreted with the urine, in which it may be detected by suitable reagents, and the time between the administration of the drug and the detection of the salicylic acid in the urine is indicative of the motor power of the stomach. These properties of salol seem to render it peculiarly suitable for testing this function. With this object in view, fifteen grains of the drug are administered in capsules or pills, and the urine examined at frequent intervals. The first appearance of salicylic acid in that fluid shows that the salol has passed into the duodenum, and has been absorbed and excreted. I mention this ingenious test for the purpose of showing the direction of present research in the diagnosis of disorders of the stomach, although I believe it can never come into general use. In the first place, the chemical test for salicylic acid in the urine is, as Stintzing[1] points out, an elaborate one, and, in the next, it is impossible for most persons to urinate at such short intervals as it demands. Again, Bourget[2] has shown by experiments on his own person that the diet is of great importance in determining the time in which salicylic acid appears in the urine. After taking nothing but meat, and a glass of water containing two per cent. of HCl, the salol test was applied and salicylic acid detected in the urine in from one hour and a quarter to one hour and a half after the repast; whereas when fruit was eaten two hours before a meal consisting of meat and vegetables, the salicylic reaction was obtained in from fifteen minutes to half an hour. These discrepancies are explained in the following manner: The fluid contained in the first portion of the small intestine is not always alkaline. On the contrary, it is often acid, its reaction depending to a large extent on the quantity of acid poured into the duodenum by the

1. *Münchener Med. Wochenschrift*, Feb. 26, 1889.
2. Loc. cit.

stomach. If to the gastric juice, already acid, there be added an additional quantity of HCl, as in the first experiment of Bourget, the chyme will have to traverse an unusually long section of intestine before it is neutralized, and the salol contained in it will be larger than usual in finding the alkaline medium in which alone it can be decomposed. On the other hand, by rendering the intestinal juice unusually alkaline, for example by eating fruit, of which the organic acids are decomposed into alkaline products, the chyme will be rapidly neutralized in the intestine, and the salol speedily decomposed.

Huber[1] proposes the following modification of the salol test: The patient takes a dose of salol, and his urine is examined on the following day at stated periods. The longer the time during which the salicylic acid is detected in the urine, the weaker is the motor power of the stomach. In certain cases of undoubted motor insufficiency, the salicylic acid reaction could be obtained on the second day after the drug had been swallowed.

Klemperer[2] recommends the following test of the motor power of the stomach on the score of greater accuracy. He injects a certain amount (100 grammes) of olive oil into the stomach, and at the end of two hours withdraws what is left. Oil, being a substance that is not absorbed by the stomach, the difference between the amount injected and that withdrawn indicates the motor power. A number of experiments proved that a healthy stomach will discharge between seventy and eighty grammes of oil out of 100 in the course of two hours. In thirteen cases of gastric catarrh, the test showed a decided diminution of motor power, the amount of oil expelled in two hours varying between twenty-three and forty-four grammes, just about half the quantity discharged in the same time in health. For obvious reasons this test is difficult of application, and Klemperer himself admits that it is not applicable in private practice.

I have now briefly alluded to the principal disorders of secretion, and have mentioned the most approved modern methods for detecting those of motor function. The third division of my subject is disorders of intervention, which can only be dealt with in the most cursory manner.

Nervous dyspepsia has acquired a permanent position in nosology since its introduction by Leube,[3] in 1879, although his conception of the affection has undergone some decided modifications. Leube supposed the chemistry of digestion to be always normal in nervous

1. *Münchener Med. Wochenschrift*, 1888, No. 19.

2. *Deutsche Med. Wochenschrift*, 1888, No. 47.

3. *Deutsches Archiv. für klin. Med.*, Bd. xxiii., 1879.

dyspepsia, whereas numerous exceptions to this rule are now recognized. He also supposed the affection to be an independent or idiopathic one, whereas it is now regarded as a neurosis which seldom finds its sole expression in the gastric and intestinal nerves, but is, in most instances, a part of a general state of neurasthenia. For the latter reason, the terms neurasthenia gastrica and neurasthenia vagosympathica have been applied to it, but nervous dyspepsia is, and doubtless will continue to be, the one most commonly employed.

The chief symptoms of nervous dyspepsia are eructations, which, unlike those of gastric catarrh, are mostly testeless and odorless, a sense of pressure and fulness in the epigastrim, and pain which is often independent of the quality of the food. A mouthful of water, for instance, will often give rise to severe pain, as is the case with a patient now under my care at the Jefferson College Hospital. As a rule, however, pain is most readily induced by fatty and acid articles of food. Pyrosis is, as already stated, a symptom of this affection, and is sometimes peculiar in being associated with a subacid condition of the secretions. A capricious appetite is a characteristic feature ot many of these cases. There may be complete anorexia alternating with periods of hyperorexia or bulimia. This morbid appetite may suddenly manifest itself at the most unseasonable hours, as in a case mentioned by Decker,[1] in his excellent article on Nervous Dyspepsia. The individual in question was constantly roused from sleep by a sense of hunger, which he was obliged to appease with a hearty meal, composed of soup, eggs, cold beef, etc.

Insomnia is a frequent symptom of this disorder, whereas, on the contrary, in quite as many cases the patient is habitually drowsy. With reference to the stools, there is nothing characteristic, except that they are not regular, a state of constipation often alternating with one of diarrhea. The diagnosis is made by the exclusion of cancer, ulcer, and catarrh. The chief difficulty consists in distinguishing this condition of neurasthenia from one of chronic catarrh; but there are certain points which, if borne in mind, will prove of great value in this differential diagnosis. In catarrh, the feelings of pressure and fulness are experienced directly after eating, and reach their maximum when digestion is at its height; whereas in nervous dyspepsia these symptoms are very variable with reference to the time of their appearance, and are often relieved by eating. They may also disappear for weeks or even months. The appetite in nervous dyspepsia is, as already stated, capricious, whereas in catarrh it is permanently absent. Valuable diagnostic aid may sometimes be derived from the

1. *Münchener Med. Wochenschrift*, May 28, 1889.

effects of treatment. For example, it is said by Decker[1] that the Carlsbad cure, which is so beneficial in cases of gastric catarrh, is invariably injurious in nervous dyspepsia.

I have now reached the last and most important part of my subject, viz., the treatment of functional disorders of the stomach, and here I must repeat what I stated with reference to their diagnosis, that the time suffices for nothing more than a few therapeutic suggestions. I will first consider the treatment of pyrosis, a symptom to which I called your attention in an early part of this paper, and I do this not only because it comes first in order, but also because its consideration will furnish some useful therapeutic hints. There are, as I stated, at least two kinds of pyrosis, one due to an excess of lactic acid and the other to an excess of hydrochloric; and the time-honored treatment of this symptom, without reference to its cause, has been the administration of sodium bicarbonate. The following experiment of Bourget shows that this drug, in the commonest form of pyrosis—that caused by an excess of lactic acid—is, at most, an evanescent palliative. To a patient with chronic gastric catarrh and dilated stomach was given a certain amount of soup, some of which was withdrawn two hours later, and found to contain 1.68 per cent. of lactic acid and no HCl. It was then exactly neutralized with sodium bicarbonate and placed in an incubator. At the end of half an hour it contained four per cent. of lactic acid. It was again neutralized and about eight grains of sodium bicarbonate added in excess. At the end of an hour free lactic acid was found. On the other hand, HCl given during a meal was found to prevent the formation of lactic acid. This corresponds with the physiological fact that the lactic acid formed during the first half hour of digestion is speedily suppressed by HCl. Bicarbonate of sodium undoubtedly neutralizes any lactic acid that may be present in the stomach, but instead of preventing its further formation, seems to increase it by creating an alkaline medium favorable to the growth of the organism of the lactic acid fermentation. Lactic acid pyrosis, therefore, is to be treated by the administration of HCl during, or immediately after, a meal.

Pyrosis hydrochlorica may be due to hyperacidity or hypersecretion. In the first instance, it is to be treated by the administration of an alkali several hours, from four to six, after a meal. In hyperacidity the gastric juice is abnormally active, its physiological work is soon performed, and then it expends its superfluous energies upon the unfortunate patient. Having waited until its work is done, it is rendered powerless for mischief by the administration of an alkali, just as in

1. Loc. cit.

peptonizing milk we boil the mixture or place it on ice to prevent the process from going too far.

The most successful treatment of hypersecretion consists in the methodical washing out of the stomach, preferably just before the principal meal, and the administration of alkalies. The latter should be given in large doses, for, as is well known, the administration of a small dose of an alkali will be followed by an increased secretion of gastric juice. Thirst is often excessive in these cases, but water should not be allowed except in small quantities, as it tends to increase the dilatation which is usually present. When the sense of thirst becomes unbearable, it is better to obtund it, as Riegel suggests, by the administration of small doses of opium than to permit the ingestion of large quantities of water. The diet should, at first, consist exclusively of albuminous substances, as carbohydrates are only partially digested, and by their fermentation aggravate the disease. Dr. Roberts, of Manchester, observing a profuse flow of saliva in cases of acid dyspepsia, and believing it to be a provision of nature for relieving the surplus acidity of the stomach, recommends the use of substances that will provoke its secretion, such as gum lozenges, containing a small amount of ginger, cayenne pepper, or pyrethrum. He has thus experienced relief in his own person and given it to others.

In cases of motor insufficiency, in addition to general hygienic measures, the vegetable bitters, especially strychnia, and alcoholic stimulants may be used with advantage. Klemperer[1] found that both substances hastened the exit of food from the stomach.

The treatment of nervous dyspepsia must vary with the cause producing it. One case will be cured by restoring the uterus to its normal position, another by curing a chronic constipation or expelling a tapeworm; still another by allaying an irritation of the spine. A restricted diet, or one of milk only, which is always beneficial in catarrh, is of little or no benefit in cases of nervous dyspepsia. General supporting measures, massage, electricity, the bromide salts, and, if anemia exists, arsenic and a mild non-constipating preparation of iron, such as the potassio-tartrate, are the chief indications. I believe also, from a limited experience of the measure, that lavage may sometimes be of decided service in soothing a hyperesthetic mucosa.

---

SMOKING early in the morning is to be strongly reprehended. Moderate smoking after the mid-day meal, or even late at night, in a healthy person is not injurious. Dr. Loomis and other observers indorse this view.

1. Loc cit.

